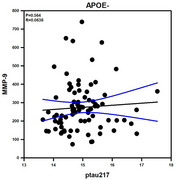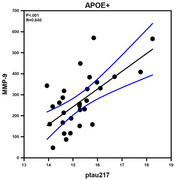# Distinct Roles of MMP‐9 and CX3CL1 on ptau217 and β‐Amyloid Levels in *APOE4* Carriers versus Noncarriers

**DOI:** 10.1002/alz70861_108571

**Published:** 2025-12-23

**Authors:** Aimee K Johnson, Carol A. Van Hulle, Hannah Zylstra, Kate Cronin, Aleshia Cole, Olivia Deering, Cecilia A. Cardenas, Kevin M. Johnson, Rachael E. Wilson, Leonardo A. Rivera‐Rivera, Richard Chapell, Carey E. Gleason, Henrik Zetterberg, Sterling C Johnson, Anthony P Auger, Cynthia M. Carlsson

**Affiliations:** ^1^ University of Wisconsin‐Madison, Neuroscience Training Program, Madison, WI USA; ^2^ University of Wisconsin‐Madison, Institute on Aging, Madison, WI USA; ^3^ Wisconsin Alzheimer's Disease Research Center, University of Wisconsin School of Medicine and Public Health, Madison, WI USA; ^4^ Division of Geriatrics and Gerontology, Department of Medicine, University of Wisconsin School of Medicine and Public Health, Madison, WI USA; ^5^ William S. Middleton Memorial Veterans Hospital, Madison, WI USA; ^6^ Wisconsin Alzheimer's Disease Research Center, University of Wisconsin‐Madison School of Medicine and Public Health, Madison, WI USA; ^7^ Wisconsin Alzheimer’s Disease Research Center, University of Wisconsin School of Medicine and Public Health, Madison, WI USA; ^8^ Department of Medical Physics, University of Wisconsin‐Madison School of Medicine and Public Health, Madison, WI USA; ^9^ Wisconsin Alzheimer's Disease Research Center, University of Wisconsin‐Madison, School of Medicine and Public Health, Madison, WI USA; ^10^ Wisconsin Alzheimer's Institute, University of Wisconsin School of Medicine and Public Health, Madison, WI USA; ^11^ Division of Geriatrics, Department of Medicine, University of Wisconsin School of Medicine and Public Health, Madison, WI USA; ^12^ Department of Biostatistics and Medical Informatics, University of Wisconsin School of Medicine and Public Health, Madison, WI USA; ^13^ Wisconsin Alzheimer's Disease Research Center, School of Medicine and Public Health, University of Wisconsin‐Madison, Madison, WI USA; ^14^ Wisconsin Alzheimer’s Institute, University of Wisconsin School of Medicine and Public Health, Madison, WI USA; ^15^ Hong Kong Center for Neurodegenerative Diseases, Hong Kong, Science Park China; ^16^ Department of Medicine, University of Wisconsin‐Madison School of Medicine and Public Health, Madison, WI USA; ^17^ Department of Neurodegenerative Disease, UCL Institute of Neurology, Queen Square, London UK; ^18^ UK Dementia Research Institute, University College London, London UK; ^19^ Clinical Neurochemistry Laboratory, Sahlgrenska University Hospital, Gothenburg Sweden; ^20^ Department of Psychiatry and Neurochemistry, Institute of Neuroscience and Physiology, The Sahlgrenska Academy, University of Gothenburg, Mölndal Sweden; ^21^ Wisconsin Alzheimer’s Disease Research Center, School of Medicine and Public Health, University of Wisconsin‐Madison, Madison, WI USA; ^22^ University of Wisconsin ‐ Madison, Neuroscience Training Program, Madison, WI USA

## Abstract

**Background:**

Fraktaline, otherwise known as CX3CL1, is a chemokine that can act on microglia to promote the release of matrix metalloproteinases (MMPs). Additionally, CX3CL1 can upregulate MMP‐9 which has been associated with alterations in memory and vasculature remodeling. MMP‐9 has also been shown to interact with tau protein, as well as APP processing, and its levels are higher in *APOE4* carriers. Therefore, we examined the role of MMP‐9 in the CX3CL1‐CX3CR1 pathway in *APOE4* carriers (APOE+) compared to noncarriers (APOE‐), as well as its implication on tau and amyloid pathology.

**Methods:**

Cerebrospinal fluid samples were collected from 116 cognitively unimpaired veterans, aged 50‐76, participating in the Brain Amyloid and Vascular Effects of Eicosapentaenoic acid (BRAVE) study (NCT02719327). We designed a custom electrochemiluminescent assay to quantify MMP‐9 levels using the Meso Scale Discovery SQ120. A NULISAseq CNS Disease Panel was used to quantify CX3CL1, ptau217, Aβ40, Aβ42 and the Roche Cobas Analyzer measured total tau.

**Results:**

In APOE‐ individuals, CX3CL1 had a direct effect on ptau217 levels (B = 0.77, SE = 0.17, *p* < .001, 95% CI [0.42, 1.11], with a standardized effect of β=0.44) with no mediating role of MMP‐9 (β = –0.0006, BootSE = 0.01, 95% CI [–0.025, 0.021]). CX3CL1 positively correlated with ptau217 (*p* <.001), Aβ40 (*p* <.001) and Aβ42 (*p* <.001). We found a negative correlation between CX3CL1 and ptau217/total tau (*p* <.001), as well as ptau217/Aβ42 (*p* =.013). MMP‐9 did not correlate with Aβ40, Aβ42, Aβ42/Aβ40, ptau217, ptau217/total tau, or ptau217/Aβ42 (*p* >0.05). However, in APOE+ carriers, MMP‐9 played a significant mediating role in the pathway involving CX3CL1 on ptau217 (β = 0.46, 95% CI [0.10, 0.75]). Importantly, MMP‐9 had a positive correlation between ptau217 (*p* <.001), CX3CL1 (*p* <.001), Aβ40 (*p* <.001) and a negative relationship with Aβ42/Aβ40 (*p* =.013) and ptau217/total tau (*p* <.001).

**Conclusion:**

We propose that APOE+ carriers experience a dysregulated CX3CL1 signaling pathway that is mediated by MMP‐9 with implications on tau regulation. Specifically, MMP‐9 is recruited into the CX3CL1 signaling pathway to reduce ptau217 phosphorylation in APOE+ carriers. If replicated, targeting MMP‐9 could be beneficial in therapeutic interventions focusing on the reduction of ptau217 pathology in neurodegenerative disorders in those who are APOE+.